# Identification and validation of prognostic and immunotherapeutic responses in esophageal squamous carcinoma based on hypoxia phenotype-related genes

**DOI:** 10.3389/fphar.2024.1344317

**Published:** 2024-03-07

**Authors:** Kai Xie, Zhe Chen, Jian Feng, Liangbin Pan, Nan Wang, Jing Luo, Yu Yao, Haitao Ma, Yu Feng, Wei Jiang

**Affiliations:** ^1^ Department of Thoracic Surgery, The Fourth Affiliated Hospital of Soochow University, Suzhou, China; ^2^ Department of Thoracic Surgery, Shanghai Chest Hospital, Shanghai Jiao Tong University, Shanghai, China; ^3^ Department of Thoracic Surgery, The First Affiliated Hospital of Soochow University, Suzhou, China; ^4^ Department of Cardiothoracic Surgery, Jinling Hospital, Medical School of Nanjing University, Nanjing, China; ^5^ Department of Respiratory Medicine, Nanjing Second Hospital, Nanjing, China

**Keywords:** esophageal squamous cell carcinoma, hypoxia, tumor microenvironment, immunotherapy, Prognosis, PKP1, PheWAS

## Abstract

The study aimed to investigate the clinical significance of the interaction between hypoxia and the immune system in esophageal squamous cell carcinoma (ESCC) microenvironment. A comprehensive evaluation of 13 hypoxia phenotype-related genes (HPRs) was conducted using data from TCGA-ESCC and two GEO cohorts. Three distinct HPRclusters were identified, and the HPRscore was established as an independent prognostic factor (*p* = 0.001), with higher scores indicating poorer prognosis. The HPRscore was validated in various immunotherapy cohorts, demonstrating its efficacy in evaluating immunotherapy and chemotherapy outcomes. Additionally, phenome-wide association study (PheWAS) analysis showed that *PKP1* had no significant correlation with other traits at the gene level. *PKP1* was identified as a potential prognostic marker for ESCC, with upregulated expression observed in ESCC patients. *In vitro* experiments showed that the knockdown of *PKP1* inhibited ESCC cell proliferation and migration. These findings suggest that the novel HPRscore and *PKP1* may serve as prognostic tools and therapeutic targets for ESCC patients.

## 1 Introduction

Esophageal cancer (EC) is a prevalent malignancy and ranks seventh in terms of global incidence and sixth in cancer-related mortality (Sung et al., 2021). Among the histological subtypes, esophageal squamous cell carcinoma (ESCC) accounts for more than 85% of cases (Huang and Yu, 2018; Wang et al., 2018). Despite recent therapeutic advancements and improved 5-year survival rates, the prognosis for ESCC patients remains unfavorable, primarily due to delayed clinical presentation and missed treatment opportunities (Rustgi and El-Serag, 2014). This highlights the urgent need for a deeper understanding of the disease and the development of effective therapeutic strategies.

Hypoxia is a common occurrence in various types of solid tumors and has significant implications for both anti-cancer treatment and the malignant progression of cancer. It is increasingly recognized that hypoxia plays a crucial role in contributing to poor prognosis (Jing et al., 2019). The rapid proliferation of cancer cells leads to a high oxygen demand, disrupting the balance between oxygen supply and consumption and resulting in the formation of an anoxic microenvironment within the tumor (Lee et al., 2020). The tissue of ESCC comprises various constituents, such as vasculature, immune cells, fibroblasts, and the extracellular matrix (Becht et al., 2016). The dysregulation of angiogenesis and accelerated cell proliferation in the tumor microenvironment frequently leads to diminished oxygen supply, thereby inducing hypoxia. Hypoxia has been shown to be associated with angiogenesis and poor prognosis in ESCC (Li et al., 2014; Yuan et al., 2023). Furthermore, immune cells play a pivotal role in regulating tumor growth through governing the invasion and metastasis of tumor cells (Lei et al., 2020). Recent studies have also revealed the influence of hypoxia on the tumor immune microenvironment (Palazon et al., 2014). However, the underlying regulatory mechanisms involving hypoxia, immunity, and ESCC remain unclear. Therefore, further studies are needed to investigate the relationship between hypoxia and immunity in ESCC.

In recent years, the application of high-throughput sequencing and public data analysis has become increasingly crucial in the discovery of biomarkers, prognosis prediction, relapse monitoring, and patient stratification (Liu et al., 2023). Several studies have employed multiple biomarkers to establish diagnostic or prognostic models in clinical settings (Xi et al., 2022; He et al., 2023). However, the role of hypoxia phenotype-related genes (HPRs) in the prognosis and response to immunotherapy in ESCC has been largely neglected.

In this study, we utilized HPRs to stratify ESCC samples based on mRNA expression levels from TCGA and GEO cohorts. Subsequently, we developed and validated a novel HPRs model and HPRscore in diverse autonomic immunotherapy cohorts, with HPRscore serving as an independent prognostic factor. Additionally, we assessed the expression and predictive impact of *PKP1* in clinical ESCC tissues. Overall, this study identifies the HPRs model and HPRscore, while also highlighting a potential therapeutic target for ESCC patients.

## 2 Results

**FIGURE 1:**
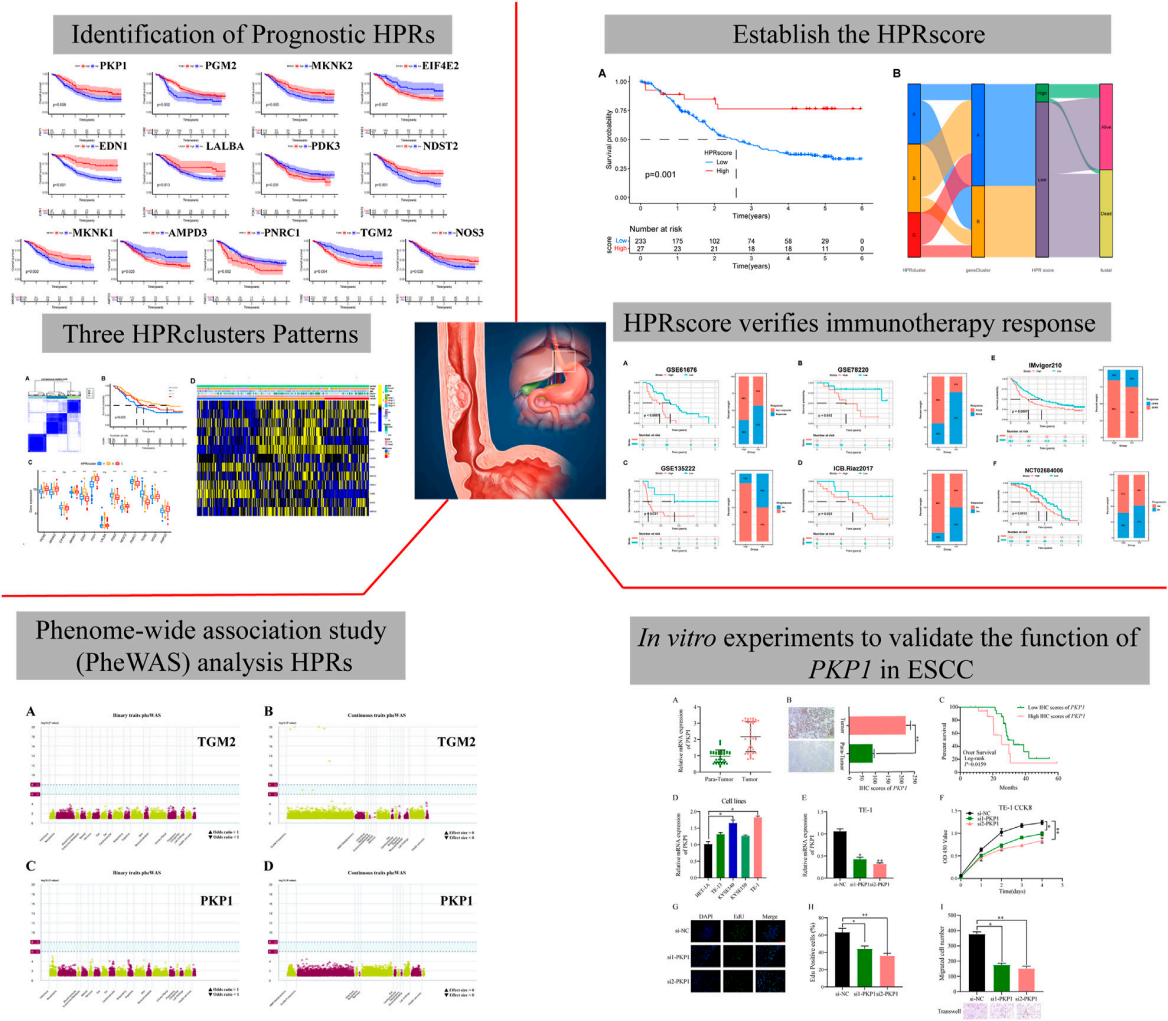
The flow diagram of the whole study. A total of 282 hypoxia phenotype-related genes (HPRs) were obtained from the KEGG and MSigDB databases. Univariate Cox combined Kaplan-Meier survival analysis was performed using data from the TCGA-ESCC and two GEO cohorts, identifying 13 independent predictors with a significance level of *p* < 0.05. Unsupervised clustering was used to identify molecular typing, which revealed three biologically unique subtypes based on the expression patterns of 13 prognostic genes. Based on this analysis, the HPR score was constructed and determined to be an independent prognostic factor (*p* = 0.001). The effectiveness of the HPRscore in immunotherapy and chemotherapy outcomes was evaluated by validating it in various immunotherapy cohorts. The potential drug targets of the HPRs and their associated side effects were explored through phenomenon association study (PheWAS) analysis. Finally, the expression and function of *PKP1* were verified *in vitro*.

Figure 1 shows the flow diagram of the whole study.

### 2.1 Expression and prognosis of HPRs in ESCC

**FIGURE 2:**
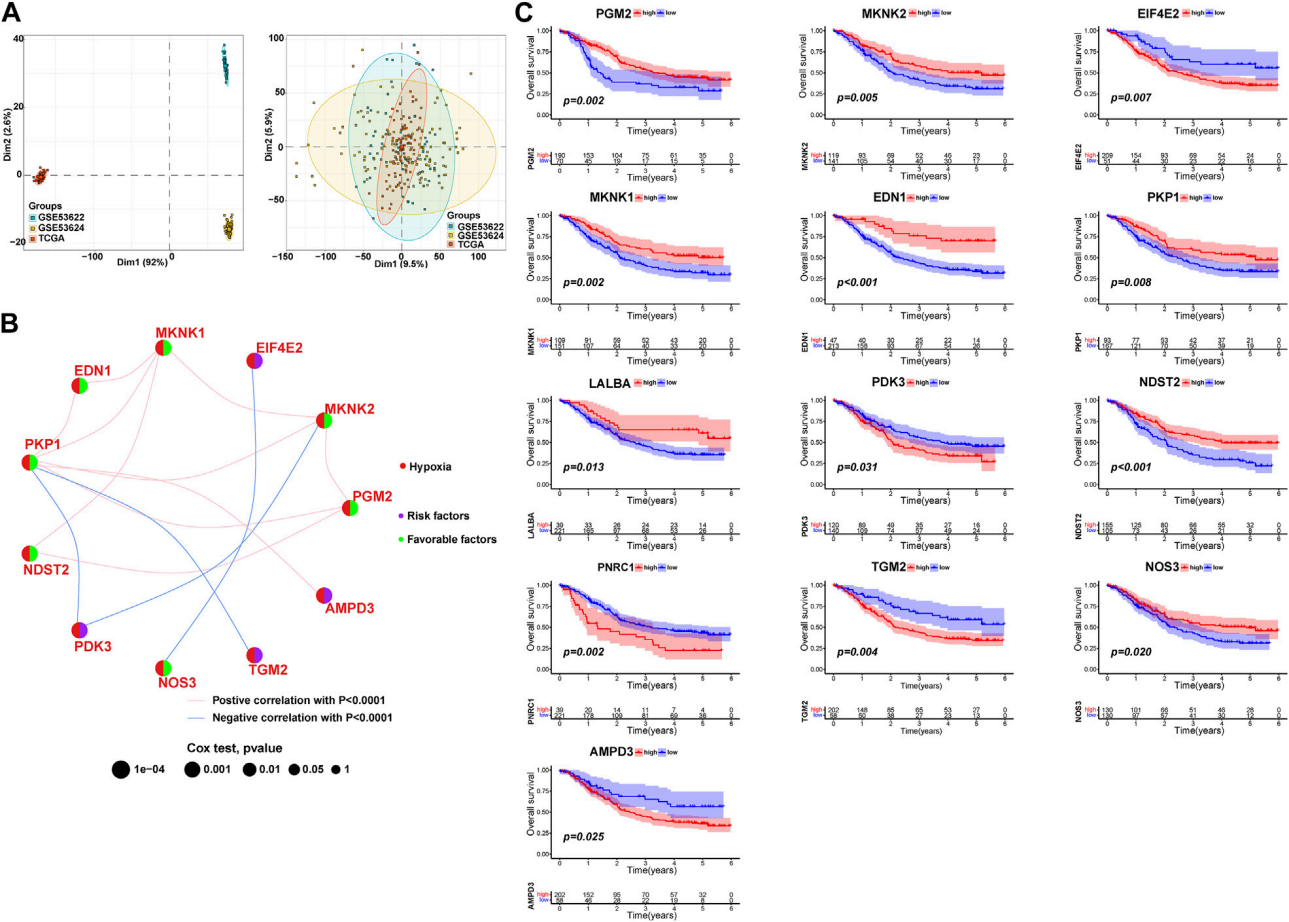
Expression and prognosis of hypoxia phenotype-related genes (HPRs) in ESCC. **(A)** Principal component analysis (PCA) showed the distribution of genes expressions in three ESCC cohorts before (left part) and after (right part) the batch effect correction. **(B)** Interaction between HPRs in ESCC. The line connecting HPRs indicated their interaction, and the thickness of the line indicated the correlation strength between HPXs. Purple and green represent negative and positive correlation, respectively. **(C)** Spearman correlation and prognostic values of hypoxia-related genes in ESCC. The circle size represents the range of significance values of each HPRs on the prognosis. The *p*-values were calculated by log-rank test. Green dots represent favorable factors for prognosis, and purple dots represent risk factors for prognosis. The lines linking HPRs represent their correlation. The thickness of the lines represents the strength of correlation between HPRs. Negative and positive correlations were marked with blue and red, respectively.

This study aimed to investigate the regulatory mechanism of HPRs in ESCC through the analysis of three independent cohorts: TCGA-ESCA (81 ESCC samples), GSE53624 (119 ESCC samples), and GSE53622 (60 ESCC samples). The combined dataset consisted of 14,047 genes and 260 ESCC samples. To effectively eliminate the batch effect across the three gene sets, PCA was conducted (Figure 2A). To identify the regulatory mechanisms underlying hypoxia phenotype-related genes in ESCC, HPRs were obtained from the KEGG *HIF-1* signaling pathway (109 genes) and the Hallmark hypoxia database (200 genes). The dataset, consisting of 282 genes, underwent merging and de-duplication (Supplementary Table S1). To demonstrate the prognostic value of HPRs in ESCC patients, univariate Cox regression and Kaplan-Meier analysis were employed with a screening threshold of *p* < 0.05 (Supplementary Table S2). Thirteen independent predictors, including *PGM2*, *MKNK2*, *EIF4E2*, *MKNK1*, *EDN1*, *PKP1*, *LALBA*, *PDK3*, *NDST2*, *PNRC1*, *TGM2*, *NOS3*, and *AMPD3*, were identified. The hypoxia network depicted the integrated landscape of HPRs interactions, regulator associations, and their prognostic value in patients with ESCC (Figure 2B). Additionally, an examination was conducted to determine the correlation between the expression levels of HPRs and patient prognosis. The results revealed that the overall survival rate of thirteen genes was statistically significant between the high expression group and the low expression group (Figure 2C).

### 2.2 HPRclusters mediated by thirteen HPRs in ESCC

**FIGURE 3:**
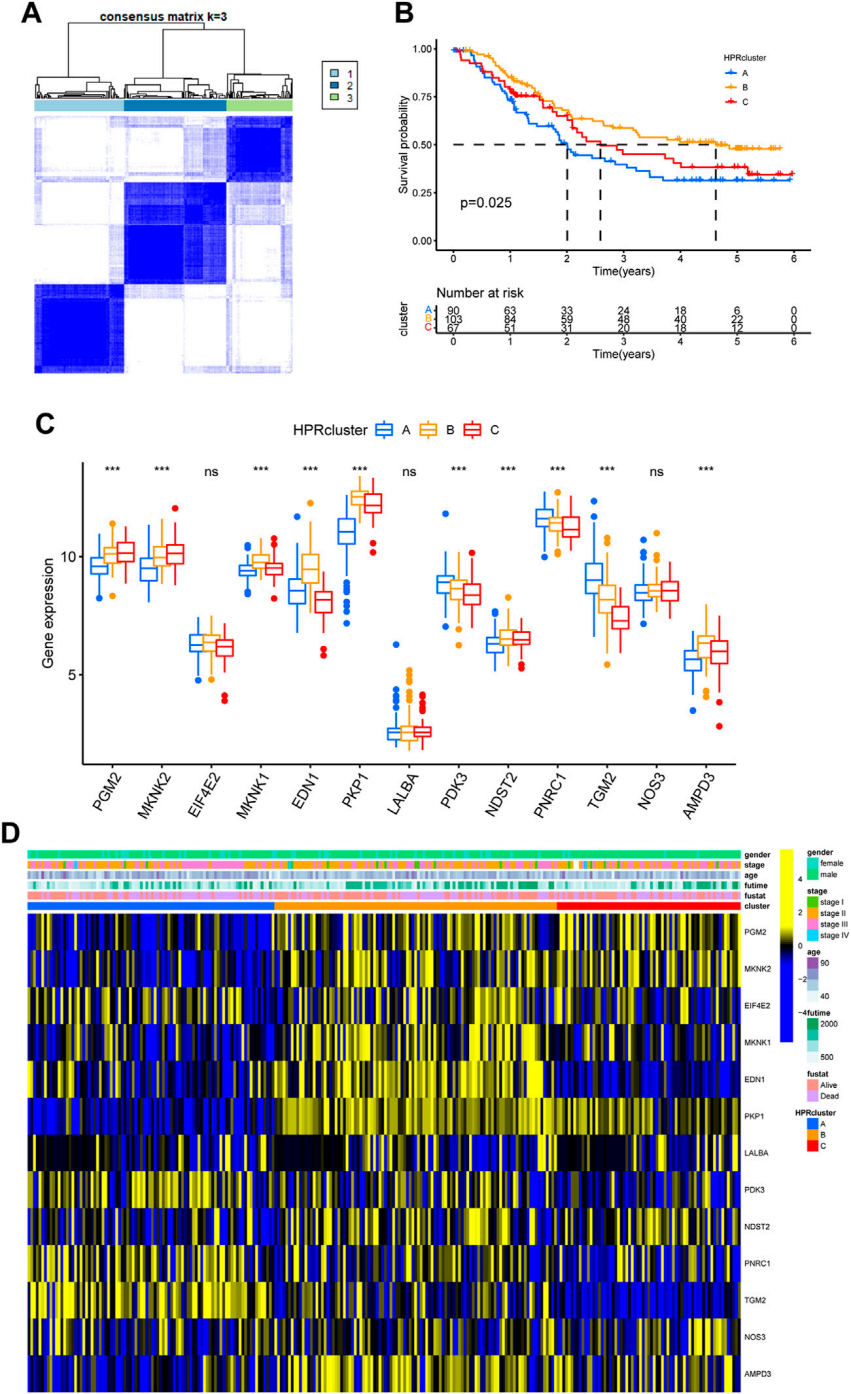
Hypoxia patterns mediated by 13 HPRs in ESCC. **(A)** The consensus matrixes for all ESCC samples displayed the clustering stability with 1,000 iterations. All samples were clustered into an appropriate number of subtypes (k = 3). **(B)** Kaplan–Meier curves showed the overall survival difference among the three HPRclusters (*p* = 0.025). **(C)** Gene expression levels of HPRs in three HPRclusters. **(D)** Differences in clinicopathologic features and expression levels of HPRs among the three HPRclusters. *p < 0.05, **p < 0.01, ***p < 0.001.

To investigate the expression characteristics of HPRs in patients with ESCC, 260 ESCC samples were analyzed using the unsupervised clustering algorithm “ConsensusClusterPlus” in the R package (Supplementary Figure S1). The clustering analysis revealed the presence of three distinct clusters: HPRcluster A (*n* = 90), HPRcluster B (*n* = 103), and HPRcluster C (*n* = 67) (Figure 3A; Supplementary Table S3). To determine the prognostic significance of these clusters, Kaplan-Meier analysis was performed. The analysis demonstrated that HPRcluster B exhibited a significant prognostic advantage (*p* = 0.025, Figure 3B), indicating that patients belonging to this cluster had a better overall prognosis compared to the other clusters. We also investigated the alterations in HPRs expression among the clusters. Figures 3C, D depicted the expression patterns, revealing that HPRs were significantly upregulated in HPRcluster B and HPRcluster C, followed by HPRcluster A. Furthermore, Fisher’s exact test was employed to examine the distribution of clinicopathologic phenotypes, including age, gender, and pathologic stage, among the clusters (Figure 3D). The results showed that HPRcluster A and HPRcluster B had a higher proportion of female patients. Additionally, patients in advanced stages (Stage III or IV) were predominantly associated with HPRcluster A. In summary, this study provides important insights into the expression characteristics of HPRs in ESCC. The identification of three distinct clusters and the observation of significant upregulation of HPRs in certain clusters, along with the prognostic advantage conferred by HPRcluster B, suggest the potential of HPRs as prognostic markers in ESCC.

**FIGURE 4:**
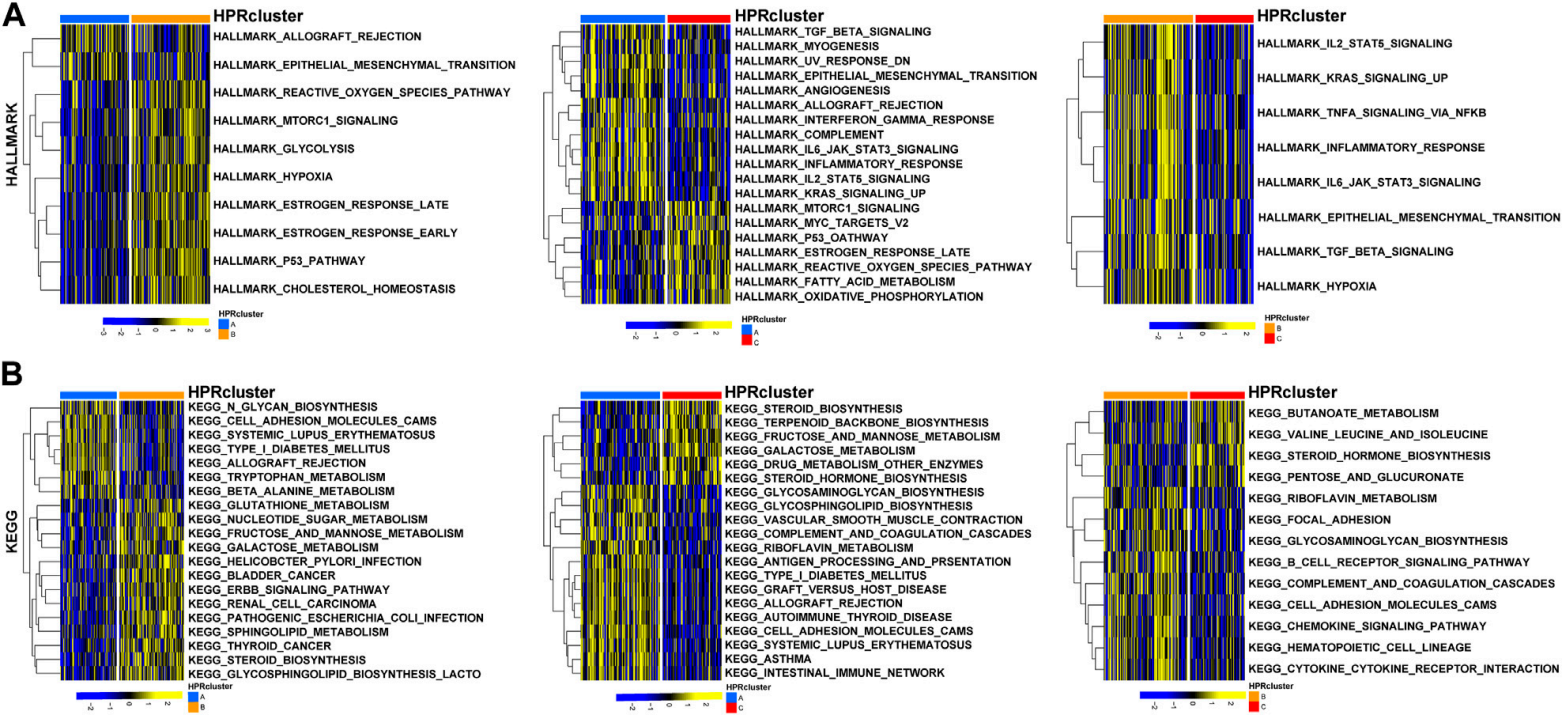
GSVA analysis. The **(A)** HALLMARK PATHWAY, **(B)** KEGG PATHWAY were downloaded separately from the Msigdb database and the pathways were scored using the R package GSVA.

To investigate the biological functionalities associated with the three clusters and their impact on prognostic outcomes, a GSVA (Gene Set Variation Analysis) enrichment analysis was conducted using the “GSVA” R package (Hänzelmann et al., 2013). Gene sets derived from the HALLMARK and KEGG pathways obtained from the MSigDB database were utilized. The GSVA enrichment analysis validated the hypothesis that the three clusters possess unique biological functionalities. In HPRcluster A, compared to HPRcluster B and HPRcluster C, significantly higher Hallmark activity was observed in *ALLOGRAFT REJECTION*, *MESENCHYMAL TRANSITION*, *OXYGEN SPECIES PATHWAY*, *MTORC1 SIGNALING*, *ESTROGEN RESPONSE LATE* and *P53 PATHWAY* (Figure 4A; Supplementary Table S4). This indicates that these biological pathways and processes are more active in HPRcluster A. Furthermore, the analysis revealed that tumors in HPRcluster A exhibited a more active *METABOLISM PATHWAY* compared to HPRcluster B, and a greater activity of *BIOSYNTHESIS PATHWAY* than HPRcluster C (Figure 4B; Supplementary Table S5). These findings provided evidence that the three clusters have distinct biological functionalities, as indicated by the enrichment analysis of various pathways. The observed differences in pathway activities among the clusters may contribute to the disparate prognostic outcomes observed in patients subjected to identical treatment protocols.

**FIGURE 5:**
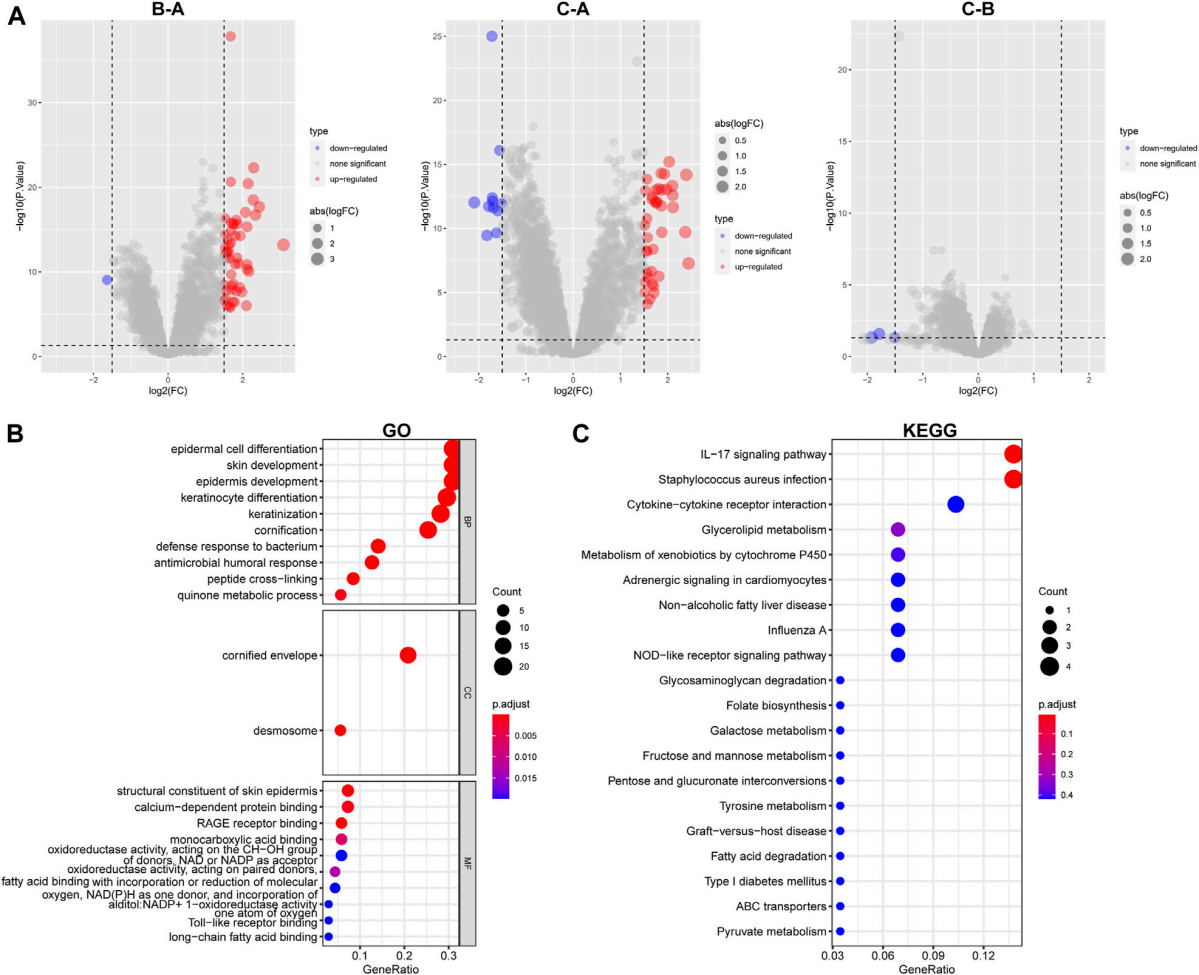
GO and KEGG analysis. **(A)** Differential analysis of the three subtypes. **(B)** GO analysis was conducted and visualized. **(C)** KEGG analysis was conducted and visualized.

In preparation for the implementation of HPRscores and the depiction of heat maps showcasing patterns of DEGs between the HPRclusters, several analyses were performed. First, pairwise comparisons of the three HPRclusters were conducted, and volcano plots were generated to visualize the DEGs. The criteria for DEG selection were set at |logFC| > 1.5 and *p* < 0.05 (Figure 5A). To identify co-expressed genes, Venn diagrams were employed, revealing a total of 77 co-expressed genes (Figure 5B; Supplementary Table S6). Subsequently, the R package “ClusterProfiler” was utilized to perform GO and KEGG enrichment analyses (Figures 5C, D). The identified genes showed significant enrichment in biological processes associated with hypoxia and immunity. This finding supported the notion that hypoxia plays a pivotal role in modulating the immune response of TME. These analyses contributed to the understanding of the gene expression patterns between HPRclusters and provide insights into the biological processes influenced by hypoxia and their impact on immune responses within the TME.

### 2.3 Different TME pattern among the three HPRclusters

**FIGURE 6:**
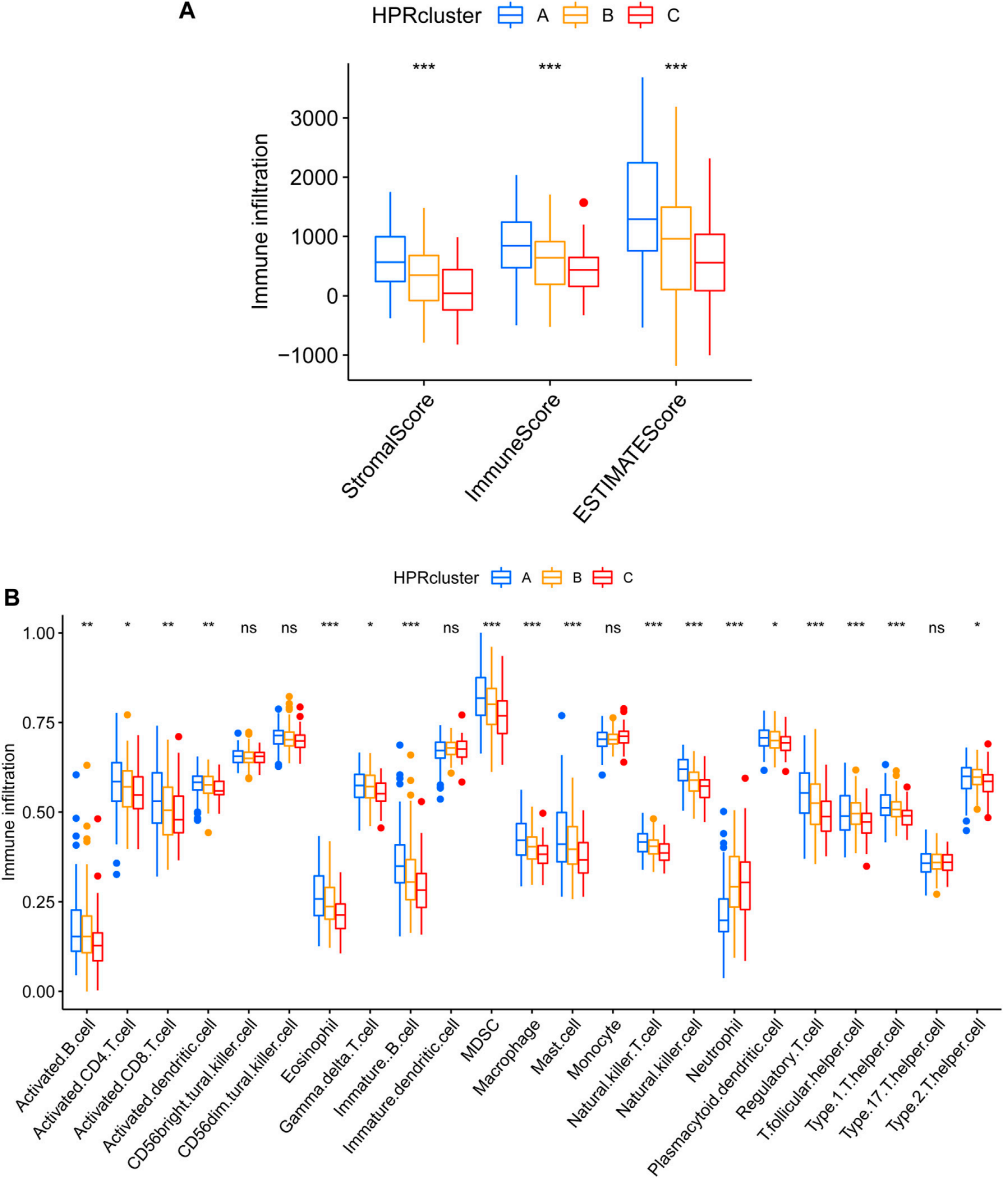
Different TME pattern among the three HPRclusters. **(A)** Differences between Stromal Score, Immune Score and ESTIMATE Score in different typologies. **(B)** Differences in immune cell infiltration between different subtypes. *p < 0.05, **p < 0.01, ***p < 0.001, ns *p* > 0.05.

After calculating the Stromal Score, Immune Score, and ESTIMATE Score for each cluster using the ESTIMATE algorithm, it was observed that HPRcluster A exhibited the highest scores in all three categories (Figure 6A). This suggested that HPRcluster A has a greater proportion of stromal cells and immune cells compared to the other clusters. The finding of higher scores in HPRcluster A is consistent with the results obtained from CIBERSORTx analysis, which showed a higher degree of infiltration by CD4 T cells, B cells, NK cells, and regulatory T cells in HPRcluster A (Figure 6B). This indicated that HPRcluster A was associated with a more pronounced immune cell infiltration, potentially reflecting a more active immune response within the tumor microenvironment.

### 2.4 Generation of HPRs signatures

**FIGURE 7:**
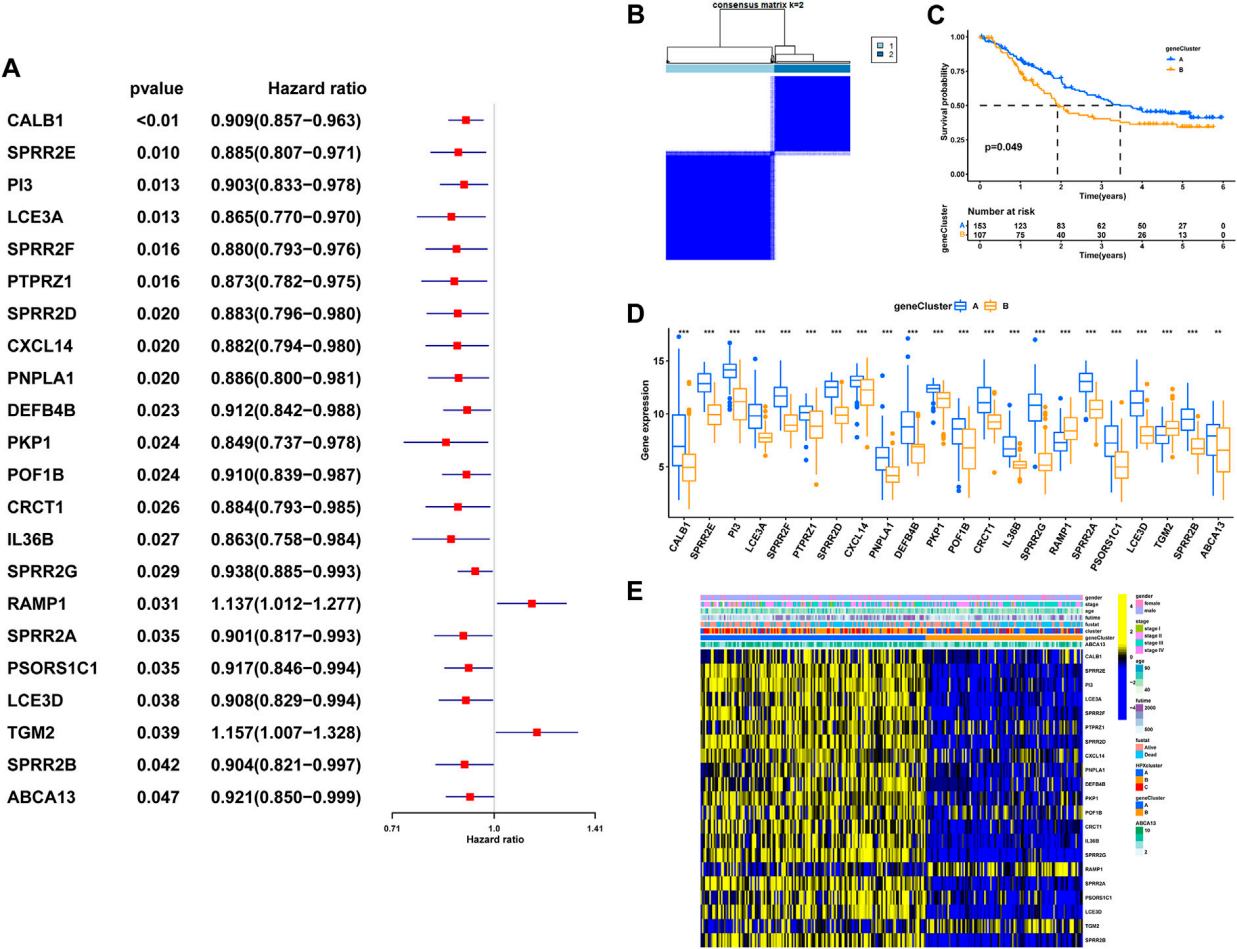
The geneCluster and its prognostic value. **(A)** Twenty-two of seventy-seven hub DEGs among the three HPRclusters demonstrated noticeable prognostic power in Cox regression. **(B)** Sub-clusters were performed with differential genes. **(C)** Survival analysis in ESCC. **(D)** Differential expression of hypoxia related genes between geneCluster. **(E)** Heatmap showing the relationship between clinical features, genes expression and sub-clusters. *p < 0.05, **p < 0.01, ***p < 0.001, ns *p* > 0.05.

To explore the potential biological attributes of HPRs, a univariate Cox regression analysis was performed on the 77 DEGs listed in Supplementary Table S7. The analysis identified 22 DEGs that were significantly associated with survival (*p* < 0.05) and were selected for further investigation (Figure 7A). Subsequently, an unsupervised clustering analysis was conducted on these 22 DEGs to classify the 260 ESCC patients into two distinct geneClusters: geneCluster A (n = 153) and geneCluster B (n = 107) (Figure 7B; Supplementary Table S8). The results revealed that patients in geneCluster B had a survival disadvantage compared to those in geneCluster A (Figure 7C, *p* < 0.049). Furthermore, notable differences in the expression of DEGs were observed between the two geneClusters. Most of the DEGs were upregulated in geneCluster A, with the exception of *RAMP1* and *TGM2*, which showed differential expression patterns (Figure 7D). Lastly, a heat map was generated to highlight the clinical characteristics of the HPRclusters and geneClusters, revealing opposing characteristics between geneCluster A and geneCluster B (Figure 7E). This visualization underscores the distinct biological attributes and potential prognostic implications associated with the two geneClusters.

### 2.5 Construction of the HPRscore and functional annotation

**FIGURE 8:**
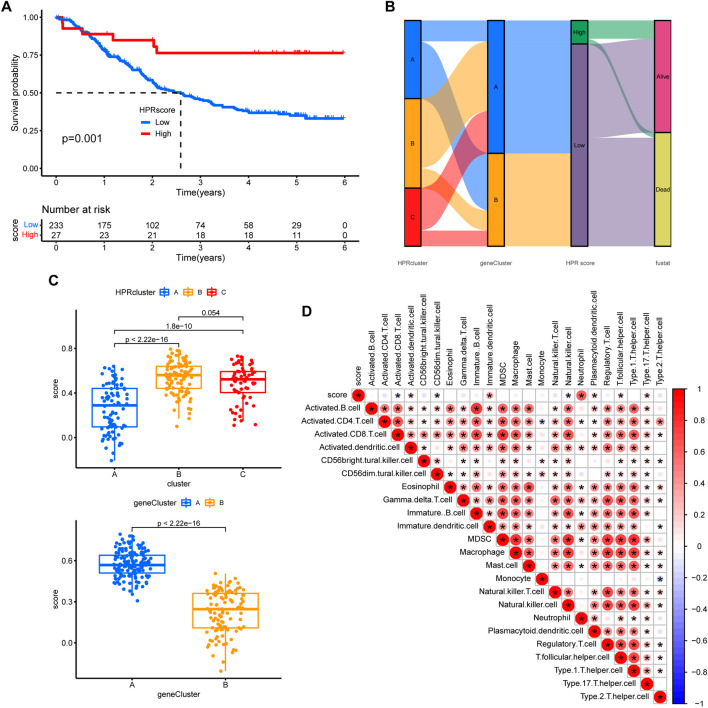
Prognostic analysis. **(A)** The overall survival of HPRscores. **(B)** Sankey diagram showing the relationship between staging, scoring and prognostic status. **(C)** Differences in geneCluster scores for HPRclusters and differences cluster scores for Twenty-two hub genes. **(D)** Correlation of immune cell infiltration. Size and color of the circle represent the Pearson correlation coefficients. *p < 0.05, **p < 0.01, ***p < 0.001, ns *p* > 0.05.

**FIGURE 9:**
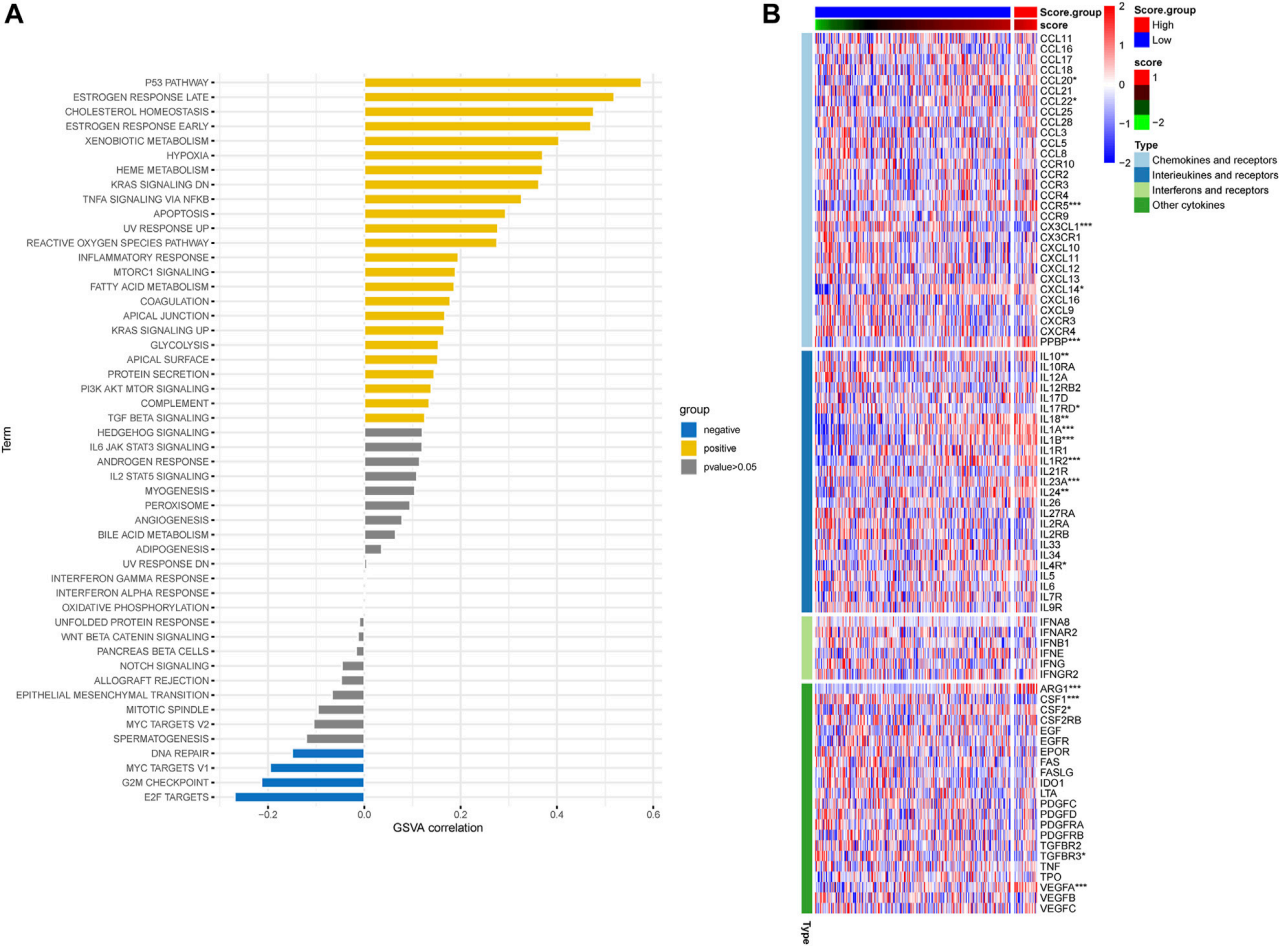
Relationship between HPRscore and immune pathways and immune-related chemokines. **(A)** The correlation between HPRscore and the activity of 50 hallmark pathways. **(B)** The immune activity and chemokine profiles in the high- and low- HPRscore groups.

In order to assess the hypoxia pattern of individual patients with ESCC, a scoring system called HPRscore was developed based on the expression of the 22 DEGs. Utilizing the R package ‘GSVA’, the 260 ESCC patients were categorized into high or low HPRscore groups using an optimal cut-off value. The prognostic value of the HPRscore was assessed through the log-rank test, which revealed that patients with a low HPRscore exhibited a poor survival outcome (*p* = 0.001, Figure 8A). An alluvial diagram was employed to visualize the changes in individual patient attributes (Figure 8B). Furthermore, the Kruskal–Wallis test was conducted and showed a significant difference in HPRscore between HPRclusters and geneClusters. HPRcluster A (Figure 8C) and geneCluster B (Figure 8C) exhibited a diminished HPRscore, and both HPRcluster A (Figure 3B) and geneCluster B (Figure 7C) demonstrated an unfavorable prognosis. Additionally, a positive correlation was observed between the HPRscore and the majority of infiltrating immune cells (Figure 8D). This suggested that higher HPRscores were associated with increased immune cell infiltration within TME. Subsequently, an analysis was conducted to determine the relationship between HPRscore and the operation of 50 hallmark pathways using GSVA. The results indicated that the HPRscore exhibited a significant correlation with inflammatory responses, hypoxia, and immune pathway signaling (Figure 9A). Further analysis of the immune activity and chemokine profiles in the high- and low-HPRscore groups revealed that the high HPRscore group was considerably enriched in chemokine-related genes, including chemokines and receptors, interleukins and receptors, interferons and receptors, and other cytokines (Figure 9B). In summary, the HPRscore had potential as a prognostic indicator for ESCC and could have significant implications for the advancement of innovative therapeutic interventions that target hypoxia and immune pathways.

### 2.6 Validation of the HPRscore and the role in predicting immunotherapeutic benefits

**FIGURE 10:**
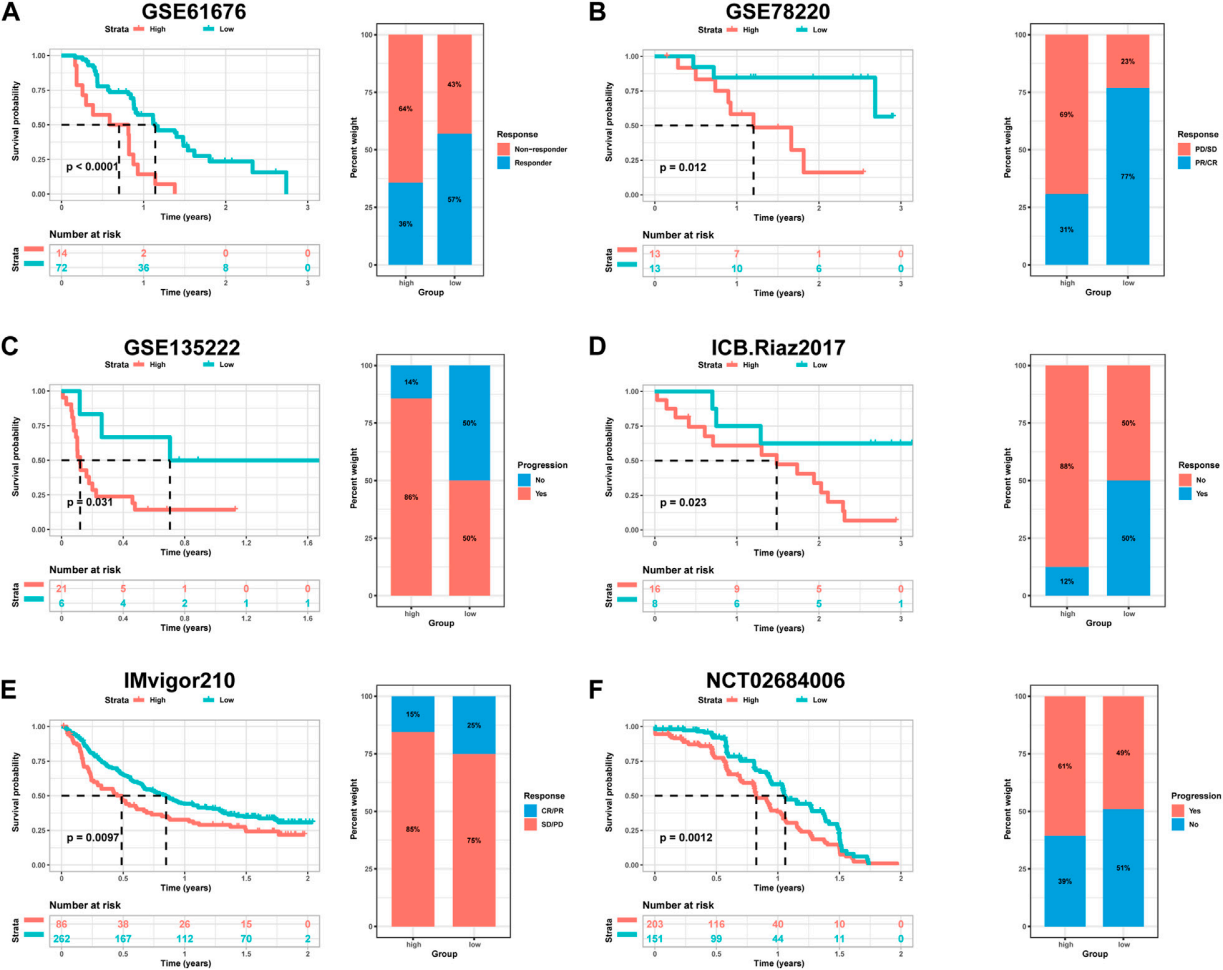
The relationship between HPRscore and predicting the benefit of immunotherapy. The relationship between **(A)** late stage non-squamous NSCLC treated with the combination of erlotinib plus bevacizumab (GSE61676), **(B)** melanoma treated with anti-PD-1 therapy (GSE78220), **(C)** advanced NSCLC treated with anti-PD-1/PD-L1 antibody (GSE135222), **(D)** melanoma treated with nivolumab therapy (ICB.Riaz 2017, GSE91061), **(E)** advanced urothelial cancer treated with anti-PD-L1 therapy (IMvigor210CoreBiologies), **(F)** advanced renal cell carcinoma treated with Avelumab (anti-PD-L1) plus axitinib *versus* sunitinib (The phase III JAVELIN Renal 101 trial, NCT02684006).

The utilization of monoclonal antibodies that block inhibitory molecules on T-cells, such as *PD-L1* and *PD-1*, has shown promise in cancer treatment, providing significant survival benefits (Curran et al., 2010). Building upon the findings that HPRscore is associated with inflammatory responses, immune pathway signaling, and chemokine-related genes, which may potentially predict the effectiveness of immunotherapy, a study was conducted to validate the accuracy of HPRscore in predicting immunotherapy efficacy using independent immunotherapy cohorts from published literature. The study included patients diagnosed with advanced non-squamous NSCLC who received a combination of erlotinib and bevacizumab. Additionally, individuals with melanoma who underwent anti-*PD-1* therapy (GSE78220, Figure 10B, *p* = 0.012), advanced NSCLC who received anti-*PD-1*/*PD-L1* antibody (GSE135222, Figure 10C, *p* = 0.031), melanoma who underwent nivolumab therapy (ICB. Riaz 2017, GSE91061, Figure 10D, *p* = 0.032), advanced urothelial cancer who received anti-*PD-L1* therapy (IMvigor210CoreBiologies, Figure 10E, *p* = 0.0097), and advanced renal cell carcinoma who were treated with Avelumab (anti-PD-L1) plus axitinib *versus* sunitinib (The phase III JAVELIN Renal 101 trial, NCT02684006, Figure 10F, *p* = 0.012) were included in the study. The results of the study indicated that patients with low HPRscore experienced significant clinical advantages and extended survival. Moreover, the immune response and favorable therapeutic outcomes observed in patients belonging to the distinct HPRscore cohort who received immune checkpoint blockade treatment were consistent with the findings. These results provided compelling support for the utilization of HPRscore as a prognostic indicator of immunotherapy effectiveness and patient prognosis.

### 2.7 Comparison of anticancer drug sensitivity between patients with different HPRscore

**FIGURE 11:**
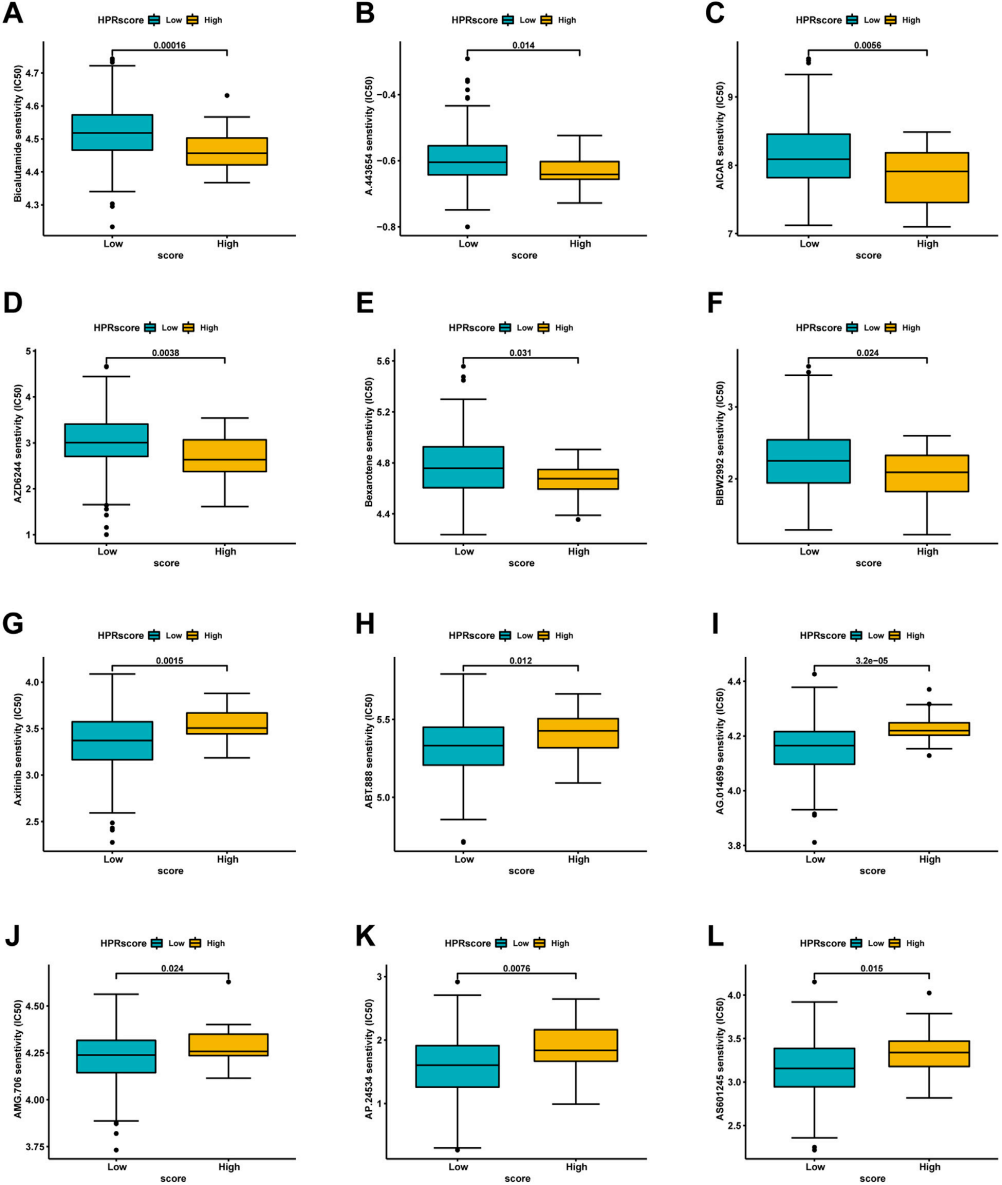
Analysis of drug sensitivity associated with HPRscore. Predicting IC50 values for multiple anti-cancer drugs. **(A–F)** High HPRscore exhibited lower IC50 values of Bicalutamide, A.443,654, AICAR, AZD6244, Bexarotene and BIBW2992. **(G–L)** Low HPRscore had significantly reduced IC50 values for non-immunotherapy agents, including Axitinib, ABT.888, AG.014699, AMG.706, AP.24534, and AS601245.

Based on the restricted efficacy of immunotherapy in managing ESCC, a tactic was implemented to identify non-immunotherapy medications and assess the vulnerability of low- and high-HPRscore subgroups. This assessment was carried out using the publicly accessible pharmacogenomics database, Genomics of Drug Sensitivity in Cancer (https://www.cancerrxgene.org), with the utilization of the R package ‘pRRophetic’. The investigation revealed notable findings. Patients with high HPRscore demonstrated lower IC_50_ values (a measure of drug potency) for the following medications: Bicalutamide, A.443,654, AICAR, AZD6244, Bexarotene, and BIBW2992 (Figures 11A–F). On the other hand, individuals with low HPRscore exhibited significantly reduced IC_50_ values for non-immunotherapy agents, including Axitinib, ABT.888, AG.014699, AMG.706, AP.24534, and AS601245 (Figures 11G–L). These findings suggested a correlation between HPRscore and drug susceptibility. In other words, the HPRscore may serve as an indicator of how susceptible patients with ESCC are to specific medications, both immunotherapy and non-immunotherapy agents.

### 2.8 PheWAS

**FIGURE 12:**
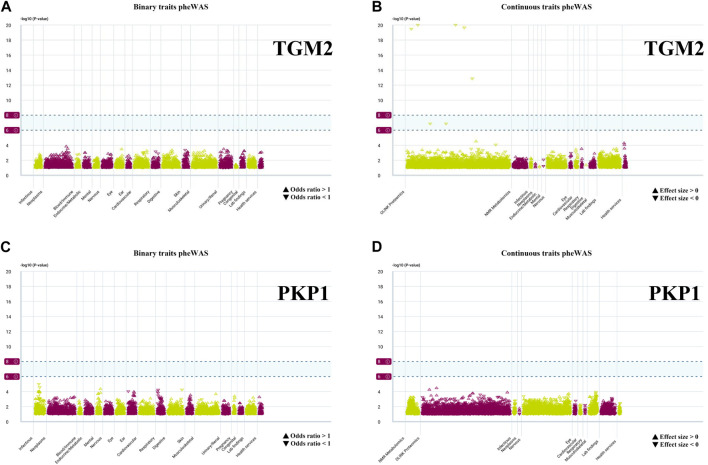
PheWAS results for each gene. **(A, B)** Binary traits and continuous traits PheWAS association with TGM2. **(C, D)** Binary traits and continuous traits PheWAS association with PKP1.

We conducted PheWAS analysis on two sets of HPR genes at the gene level, as depicted in Figures 2C, 6A, using a dataset of 17,361 binary phenotypes and 1,419 quantitative phenotypes obtained from the AstraZeneca PheWAS portal database. PheWAS results provide insights into associations between genetically determined protein expression and specific diseases or traits. Except for *TGM2*, *AMPD3*, *PSORS1C1*, *POF1B*, *NOS3*, and *PSORS1C1*, no other genes exhibited significant associations with traits at the gene level, based on the predefined significance threshold (*P* < 1E−8) (Supplementary Table S9). This suggests the possibility of potential side effects and horizontal pleiotropy affecting these genes, which may impact drug targeting strategies for these gene targets. Notably, the simultaneous occurrence of the *TGM2* and *PKP1* genes in both sets piqued our interest for further exploration. PheWAS results indicated that *TGM2* was primarily associated with Cardiometabolic traits, suggesting that drugs targeting the *TGM2* gene in ESCC may have an impact on these traits (Figures 12A, B). On the other hand, *PKP1* did not show significant associations with other traits at the gene level (Figures 12C, D). Consequently, *PKP1* was selected and validated for further investigation.

### 2.9 The expression and prognostic value of *PKP1* were evaluated in ESCC samples

*PKP1*, as a HPR gene, has been documented to exhibit aberrant expression in various cancers (Wang et al., 2020; Boyero et al., 2022). Nevertheless, its involvement in ESCC remains inadequately investigated. *PKP1* expression was significantly downregulated in esophageal squamous cell lines under hypoxia, suggesting that our analysis was reliable (Supplementary Figure S2). The mechanism may be related to the hypoxia inducible factor (*HIF*) signaling pathway or under hypoxia conditions, some mirnas bind to *PKP1* mRNA, inhibit its translation or degradation, and lead to downregulation of its expression.

**FIGURE 13:**
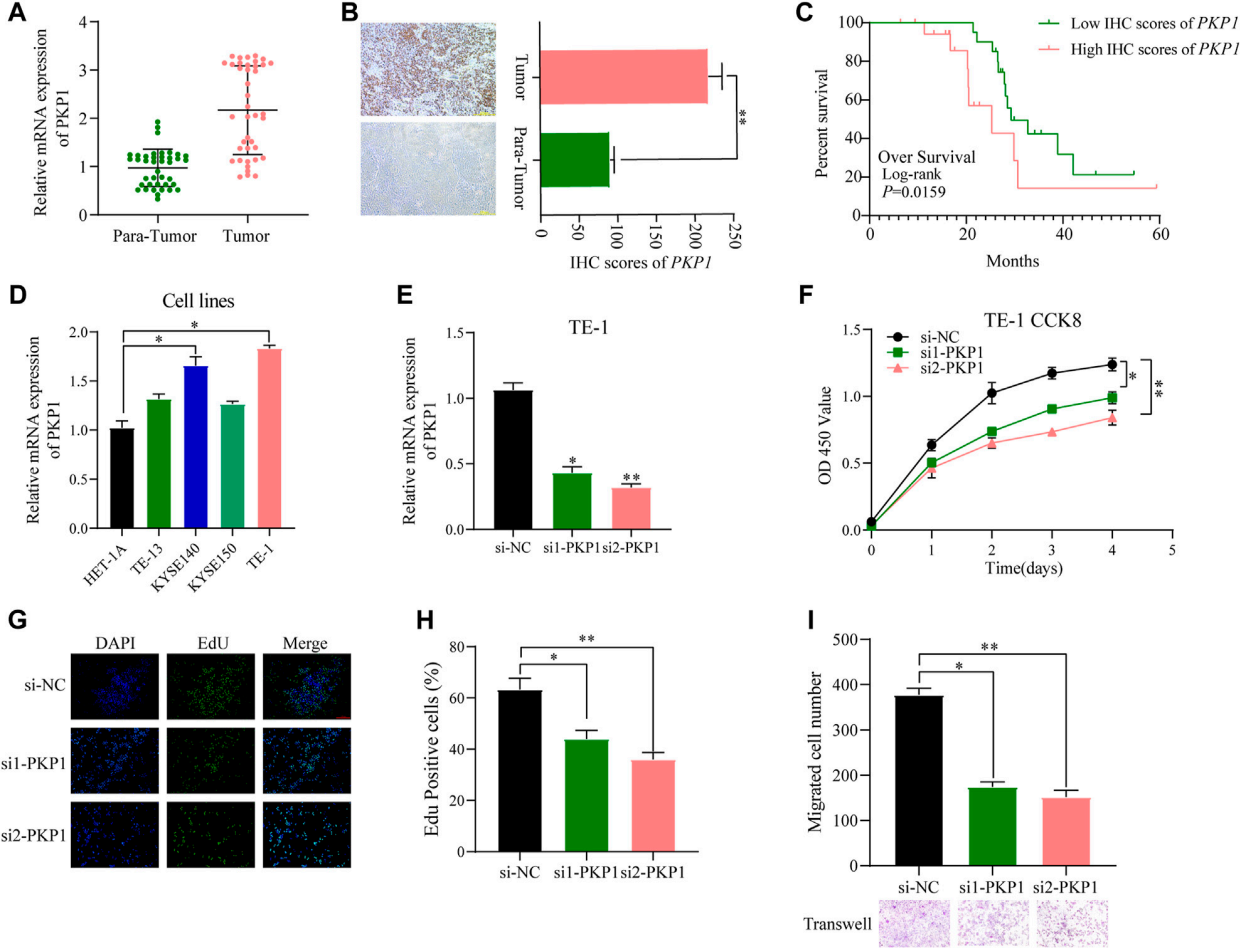
Evaluation of *PKP1* expression levels and prognostic value in esophageal samples. **(A, B)** The mRNA and protein expression of *PKP1* was significantly upregulated in ESCC compared with tissues adjacent tissues. **(C)** Kaplan–Meier survival analysis of over survival according IHC scores of *PKP1* in 40 ESCC patients. **(D)** The mRNA level expression of *PKP1* in ESCC cell lines. **(E)** Validation of siRNA knockdown efficiency in TE-1 cells. **(F–I)** Transfection of siRNA into TE-1 cells and CCK-8 **(F)**, EdU **(G, H)**, and Transwell **(I)** assay detected the cell proliferation and migration. *p < 0.05, **p < 0.01.

To further ascertain the potential clinical significance of *PKP1* in ESCC, we conducted qRT-PCR and IHC analyses to investigate the expression levels of *PKP1* in ESCC tissues and adjacent tissues. The results showed a significant upregulation of *PKP1* expression in ESCC tissues compared to adjacent tissues (Figures 13A, B). Based on the IHC scores, patients were divided into two groups: high *PKP1* expression and low *PKP1* expression. The analysis revealed a significant association between elevated *PKP1* expression and various clinicopathological characteristics of ESCC, including tumor size (*p* = 0.0267), invasion depth (*p* = 0.0016), lymph node metastasis (*p* = 0.0251), and clinical stage (*p* = 0.0102) (Table 1). Additionally, Kaplan-Meier survival analysis indicated that patients with higher *PKP1* expression had a notable decrease in overall survival (*p* = 0.0159) (Figure 13C), suggesting that *PKP1* may serve as a prognostic marker in ESCC.

**TABLE 1:** Correlation between *PKP1* expression and clinicopathological features in ESCC patients (*n* = 40).

**Clinicopathological parameters**	**Numbers**	**IHC scores of *PKP1***	***p*-value^a^**	
		**Low scores**	**High scores**	
Gender				
Male	22 (55%)	9	13	0.2036
Female	18 (45%)	11	7	
Age (year)				
<60	25 (62.5%)	14	11	0.3272
≥60	15 (37.5%)	6	9	
Tumor size				
<5 cm	19 (47.5%)	13	6	0.0267*
≥5 cm	21 (52.5%)	7	14	
Location				
Upper	5 (12.5%)	3	2	0.8854
Middel	23 (57.5%)	11	12	
Low	12 (30%)	6	6	
Invasion depth				
pT 1	15 (37.5%)	13	2	0.0016*
pT 2	11 (27.5%)	3	8	
pT 3	14 (35%)	4	10	
Lymph node metastasis				
Positive	23 (57.5%)	15	8	0.0251*
Negative	17 (42.5%)	5	12	
Differentiation				
Poor	7 (17.5%)	2	5	0.0816
Moderate	28 (70%)	12	14	
Well	7 (17.5%)	6	1	
Clinical stage*				
I	9 (22.5%)	7	2	0.0102*
II	16 (40%)	10	6	
III	15 (37.5%)	3	12	

* *p* < 0.05.

^a^ Chi-square test.

Compared with normal cells, the expression of *PKP1* was significantly upregulated in ESCC cell lines (KYSE140 and TE-1 cell lines) (Figure 13D). To understand the functional role of *PKP1*, its expression was modulated using siRNA-mediated knockdown (Figure 13E). The knockdown of *PKP1* expression resulted in a significant inhibition of cell proliferation and migration, as demonstrated by CCK-8, EdU, and Transwell experiments (Figures 13F–I). These findings suggest that *PKP1* plays a role in promoting cell proliferation and migration in ESCC cells.

## 3 Discussion

ESCC is known to be challenging to treat effectively, and current treatment options have limited success (Codipilly et al., 2018). Immunotherapy, although promising, has shown limited response rates in ESCC patients (Luo et al., 2021; Sun et al., 2021; Doki et al., 2022; Wang et al., 2022). Therefore, there is a need for innovative treatment strategies to improve outcomes.

In recent times, there has been an increased focus on the contribution of TME to the pathogenesis, advancement, and sustenance of diseases. The TME comprises a diverse range of constituents, such as immune cells, stromal cells, chemokines, and cytokines, which can synergize to establish a persistent inflammatory, immunosuppressive, and pro-carcinogenic milieu, thereby evading immune surveillance and bolstering tumor cell viability (Wu and Dai, 2017). Additionally, hypoxia within the tumor microenvironment can modulate gene expression, foster cell survival, and augment resistance to apoptosis induction (Jing et al., 2019). Furthermore, it is postulated that tumor cells enclosed within a hypoxic TME exhibit heightened aggressiveness and resistance to pharmaceutical interventions (Qian and Rankin, 2019). Ye et al. have categorized tumor specimens into high and low hypoxia score groups, identified molecular modifications associated with anticancer drug responses, and illustrated the influence of hypoxia in fostering tumor heterogeneity and viability (Ye et al., 2019). Bhandari et al. have conducted a quantitative analysis of hypoxia across multiple cancer types, revealing a positive correlation between heightened hypoxia and mutational burden (Bhandari et al., 2020).

The study focused on investigating the role of HPRs in ESCC regarding their biological function, prognostic value, correlation with TME, immunotherapy response, and chemotherapy resistance. Through analyzing genetic expression and prognosis in ESCC patient cohorts, we identified three distinct hypoxia clusters characterized by different gene expression patterns. Among these clusters, HPRcluster A exhibited the highest scores for stromal and immune cell infiltration, suggesting a more favorable immune response. The hypoxic microenvironment in tumor cells promotes glucose uptake, which can impact the functionality of crucial immunologically active cells. The high-risk subset showed increased infiltration of macrophages and T cells, known to inhibit the effectiveness of immune checkpoint inhibitor treatment (Samanta and Semenza, 2018). It has been observed that tumor cells have a superior ability to utilize glucose as an energy source compared to T cells, leading to competition for this vital resource (Chang et al., 2015). Consequently, T cells experience hindered nutritional metabolism. Hypoxia intensifies glucose metabolism pathways and enhances glucose absorption by tumor cells, creating an unfavorable nutritional state for T cells, impairing their immune functions and ability to eliminate tumor cells. Additionally, the heightened glycolytic activity of tumor cells in a hypoxic environment generates an acidic microenvironment that further affects T cell functionality (Leone and Powell, 2020). HPRcluster A was identified as an immunoinflammatory phenotype, characterized by the infiltration of adaptive immune cells and immune activation, which correlated with an unfavorable prognosis. Consistent with the clustering results of HPRs, two genomic subtypes associated with immune activation were identified, supporting the crucial role of hypoxia in immune regulation within the TME.

To address individual heterogeneity, we developed a novel scoring system, HPRscore, to evaluate and quantify the hypoxia response pattern in patients with ESCC. Our findings revealed that patients with a low HPRscore experience unfavorable survival outcomes. Interestingly, the low HPRscore group showed enrichment in pathways associated with immune activation, indicating an immune-inflamed phenotype. In contrast, the high HPRscore group was enriched in pathways related to stromal components, suggesting an immune-excluded and immune-desert phenotype. These results were further validated in the IMvigor210 cohort, with the immune-desert and excluded phenotypes exhibiting higher HPRscores, while the immune-inflamed phenotype displaying significantly lower HPRscores.

Furthermore, we have demonstrated the prognostic significance of the HPRscore in relation to checkpoint blockade therapy across multiple patient cohorts. Our analysis included six distinct cohorts, encompassing patients with advanced non-squamous NSCLC treated with erlotinib and bevacizumab (Baty et al., 2017), melanoma patients receiving anti-PD-1 therapy (Hugo et al., 2016), patients with advanced NSCLC treated with anti-PD-1/PD-L1 antibody (Kim et al., 2020), melanoma patients receiving nivolumab therapy (Riaz et al., 2017), patients with advanced urothelial cancer treated with anti-PD-L1 therapy (Mariathasan et al., 2018), and patients with advanced renal cell carcinoma receiving Avelumab (anti-PD-L1) in combination with axitinib or sunitinib (Motzer et al., 2020). The results consistently demonstrated that patients with a low HPRscore experience greater clinical benefits from checkpoint blockade therapy compared to non-responders.

## 4 Conclusion

In summary, this study contributes to our understanding of the role of hypoxia-related genes (HPRs) and the TME in ESCC. The HPRscore has potential clinical utility as a prognostic tool and treatment guide, particularly in the context of immunotherapy. These findings may pave the way for personalized approaches in ESCC management and the development of novel therapeutic interventions. Further research is warranted to validate and expand upon these findings for the benefit of ESCC patients. And *PKP1* may be a potential therapeutic target for ESCC.

## 5 Materials and methods

### 5.1 Data collection and preprocessing

The present study analyzed RNA expression in ESCC by utilizing data from two databases, namely, The Cancer Genome Atlas (TCGA; https://portal.gdc.cancer.gov/) and the Gene Expression Omnibus (GEO; https://www.ncbi.nlm.nih.gov/geo/). RNA expression data were derived from the TCGA cohort as well as two GEO cohorts (GSE53624 and GSE53622), which were subjected to background adjustment and quantile normalization of the raw “CEL” files. The “combat” algorithm was utilized to address potential batch effects, and it involved the use of the limma and sva R packages (Leek et al., 2012; Ritchie et al., 2015). The differential expression of mRNAs was determined based on a false discovery rate (FDR) of less than 0.05 and an absolute log_2_ fold change (|log_2_FC|) of at least 1, with the use of R 4.1.1 software and the limma package.

Hypoxia phenotype-related genes (HPRs) were identified from two distinct data-bases, the KEGG database (https://www.kegg.jp/pathway/hsa04066) and the Molecular Signatures Database (MSigDB; http://www.gsea-msigdb.org/gsea/msigdb/cards/HALLMARK_HYPOXIA). More specifically, we curated a list of 109 HPRs associated with the *HIF-1* signaling pathway using the KEGG database. Additionally, we included the Hallmark hypoxia gene sets (n = 200) from the MSigDB database (Liberzon et al., 2015). For the complete inventory of these genes, please refer to Table S1.

### 5.2 Unsupervised clustering based on HPRs

Unsupervised cluster analysis was conducted to distinguish exclusive hypoxia modification patterns and stratify patients for further examination based on the ex-pression levels of thirteen HPRs. The R software package “ConsensusClusterPlus” was utilized for the analysis with 1,000 iterations to guarantee the stability of the clustering results. The optimal number of clusters was determined using the consensus clustering algorithm (Wilkerson and Hayes, 2010).

### 5.3 Generation of the HPRs signature

To assess the hypoxia modification pattern in ESCC patients, we developed a novel scoring system called HPRscore. This scoring system utilizes a hypoxia gene signature. Initially, we identified differentially expressed genes (DEGs) from individual HPRclusters and standardized them across all ESCC samples. We then performed unsupervised cluster analysis on the overlapping genes to categorize patients into distinct groups for further analysis. The consensus clustering algorithm helped determine the number and stability of gene clusters. Using univariate Cox regression analysis, we identified a prognostic gene within the signature. Principal component analysis (PCA) was then conducted to establish the HPR signature, with the signature scores derived from the main components 1 and 2.

### 5.4 Estimating of immune infiltration

Single-sample gene-set enrichment analysis (ssGSEA) was utilized to determine the activity levels of specific biological pathways or cell types in individual samples based on their gene expression profiles (Hänzelmann et al., 2013). We applied ssGSEA to assess and quantify immune infiltration in each sample using previously researched immune cell marker gene expression information by Charoen-tong (Charoentong et al., 2017). The enrichment score obtained via ssGSEA represented the relative abundance of infiltration for each immune cell. Additionally, we used the “ESTIMATE” package to calculate ImmuneScore, StromalScore, and ESTIMATEScore. ImmuneScore provided an estimate of immune cell infiltration within the tumor microenvironment, while Stro-malScore indicated the abundance of stromal cells. ESTIMATEScore combined both ImmuneScore and StromalScore, which provided an overall estimate of tumor purity.

### 5.5 Gene set variation analysis

The R package “GSVA” was used to perform enrichment analysis and investigate differences in biological processes among the HPR subtypes (Hänzelmann et al., 2013). Gene set variation analysis (GSVA), a non-parametric and unsupervised method, was employed to assess pathway and biological process activities across different expression datasets. For the GSVA analysis, gene sets including Gene Ontology (GO) and KEGG were obtained from the MSigDB database. To visually represent hypoxia-related pathways, heatmaps were generated, highlighting pathways with a significance level of *p* < 0.05.

### 5.6 The HPRscore generation process

The aim of study was to establish a customized scoring system for assessing hypoxia levels in individual patients with ESCC. We developed this scoring system through a series of steps, which began with normalizing DEGs from different hypoxia clusters across all samples and identifying overlapping genes. We identified 77 common differential genes through differential analysis and Venn diagrams among the three HPRclusters. Next, we performed univariate Cox regression analysis for each gene and 22 genes with significant prognostic value for further analysis. The hypoxia score (HPRscore) was calculated using the “GSVA” R package (Hänzelmann et al., 2013). Using the expression data for HPRclusters, we computed HPRscore through PCA using the formula:

$$\text{HPRscore}=\sum \left(\text{PC}1_{i}+\text{PC}2_{i}\right),$$

where ‘i' represents the expression of HPRs. This customized scoring system has great potential for individualized hypoxia evaluation and prognostic prediction improvement in ESCC patients.

### 5.7 Phenome-wide association study (PheWAS) analysis

To assess potential drug targets and their associated side effects, PheWAS was conducted using the AstraZeneca PheWAS Portal (https://azphewas.com/) (Wang et al., 2021; Dhindsa et al., 2023). AstraZeneca PheWAS Portal is a repository of gene-phenotype associations for phenotypes derived from electronic health records, questionnaire data, and continuous traits computed on exomes released by UK Biobank. All genomic coordinates in this Portal are based on GRCh38. Continuous phenotypes are rank-based inverse normal transformed before analysis. To mitigate false positives, we applied multiple corrections and set a significance threshold of 1E-8, following the default setting in the AstraZeneca PheWAS Portal.

### 5.8 Patient tissue samples

A total of forty pairs of ESCC and adjacent normal tissues were procured from pa-tients who underwent surgery at the First Affiliated Hospital of Soochow University and received a pathological diagnosis. This study was carried out with the explicit in-formed consent of all patients and received approval from the Ethics Committee of the First Affiliated Hospital of Soochow University.

### 5.9 Cell culture

ESCC cell lines (TE-1, TE-13, KYSE150, KYSE140) and normal human esophageal epithelial cell line (HET-1A) were obtained from the Shanghai Institutes for Biological Sciences (Shanghai, China). These cell lines were cultured in 1,640 medium (supplemented with 10% fetal bovine serum (FBS) (KeyGene, Nanjing, China) and 1% penicillin-streptomycin) at 37°C in a humidified 5% CO_2_ atmosphere.

### 5.10 RNA extraction and quantitative real-time PCR (qRT-PCR)

Total RNA was extracted using the TRIzol reagent (Invitrogen, Carlsbad, CA) following the manufacturer’s instructions. The extracted RNA was then reverse transcribed into complementary DNA by synthesis kit (Takara, Cat: RR036A, KeyGEN). The qRT-PCR experiment was performed in triplicate and the data were normalized to *β-actin* using the 2^−ΔΔCT^ method. The primer sequences used for the qRT-PCR analysis were as follows: *ACTIN* forward, 5′-GTC​ATT​CCA​AAT​ATG​AGA​TGC​GT-3′; *ACTIN* reverse, 5′-GCA​TTA​CAT​AAT​TTA​CAC​GAA​AGC​A-3′; *PKP1* forward, 5′-TCA​GCA​ACA​AGA​GCG​ACA​AG-3′; *PKP1* reverse, 5′-TCA​GGT​AGG​TGC​GGA​TGG-3′.

### 5.11 siRNA construction and cell transfection

The siRNAs that targeted *PKP1* were obtained from RiboBio (Guangzhou, China). The transfection of these siRNAs into cells was performed using Imax (Invitrogen, Carlsbad, CA, United States) in accordance with the guidelines provided by the manufacturer. The siRNA sequences as follows: si1-*PKP1* sense sequence, 5′-GGC​UGA​CAA​UUA​CAA​CUA​Utt-3′; si1-*PKP1* antisense sequence, 5′-AUA​GUU​GUA​AUU​GUC​AGC​Caa-3′; si2-*PKP1* sense sequence, 5′-GCU​UUG​CCG​UCG​GAC​CAA​Att-3′; si2-*PKP1* antisense sequence, 5′-UUU​GGU​CCG​ACG​GCA​AAG​Cca-3′; si-NC sense sequence, 5′-UAA​CGA​CGC​GAC​GAC​GUA​Att-3′; si-NC antisense sequence, 5′-UUA​CGU​CGU​CGC​GUC​GUU​Att-3′.

### 5.12 CCK-8 and EdU assay

After transfection and incubation for 24 h, the cells were inoculated into 96-well plates at a density of 2000 cells per 100 μL. To ensure reproducibility, the same sample was placed in 5 repeated wells. The cells were then incubated at 37°C for 6 h to allow them to attach to the well walls. Next, 10 μL of CCK-8 was added to each well, and the baseline absorbance at 450 nm was recorded. Subsequently, the absorbance at 450 nm was measured every 24 h for a total of 4 days.

EdU cell proliferation staining was conducted utilizing an EdU kit (Cat.C10310-3, Ruibo, China), following the guidelines provided by the manufacturer. To be specific, 1.0×10^5^ cells per 100 μL were inoculated in a 96-well plate. To prepare the appropriate amount of 50 μM EdU medium, the EdU solution (reagent A) was diluted in a ratio of 1,000:1 with cell complete medium. Next, 100 μL of the 50 μM EdU medium was added to each well, and the cells were incubated for 2 h. After incubation, the medium was discarded, and the cells were washed twice with PBS for 5 min each time. Subsequently, 50 μL of cell fixative was added to each well and incubated at room temperature for 30 min. Following the incubation, the fixative was discarded, and 50 μL of 2 mg/mL glycine was added to each well. The plate was then incubated in a decolorized shaker for 5 min. The glycine solution was subsequently discarded, and the cells were washed twice with PBS for 5 min each time. Next, 100 μL of PBS containing 0.5% Triton X-100 was added to each well and incubated in a shaker for 10 min. The cells were then rinsed with PBS for 5 min. Each well was stained with 100 μL of 1 Apollo solution and 100 μL of 1 Hoechst 33,342 solution, respectively. Finally, after washing with 100 μL of PBS three times, the images were observed under a fluorescence microscope, and the proliferation rate was calculated.

### 5.13 Transwell assays

The Transwell experiment was conducted using a 24-well plate with a Transwell insert featuring an 8 μm pore size. The upper cavity of the Transwell insert was supplemented with 300 μL of serum-free medium containing 2.5×10^4^ cells, while the lower cavity was supplemented with 700 μL of medium containing 10% fetal bovine serum. The plate was then incubated in an incubator for 24 h. After incubation, the Transwell chamber was removed, and the culture solution was discarded. To remove any remaining matrix glue and cells from the chamber, a PBS-soaked cotton swab or cotton was used to gently wipe the surface. The cells were then fixed with 4% methanol for 30 min and washed with PBS for 5 min. Subsequently, the cells were stained with crystal violet for 10 min, followed by three washes with PBS. After drying, the cells were photographed under a microscope in three to five fields, and the average was quantified using ImageJ.

### 5.14 Immunohistochemistry (IHC)

The tissue slices were incubated in a 65 incubator for 1 h and then soaked in xylene for three 10-min intervals. Subsequently, they were placed in anhydrous ethanol, followed by 95%, 90%, and 80% ethanol, each for 5 min. The slices were then washed twice with PBS for 5 min each time. To inactivate endogenous peroxidase activity, the slices were incubated with 3% H_2_O_2_ deionized water for 10 min. After another two washes with PBS for 5 min each time, the slices were boiled in 0.01 M citric acid buffer (pH = 6.0) at 95 for 15–20 min. The slices were then rapidly cooled in cold water to room temperature for 15 min, followed by two additional washes with PBS for 5 min each time. To prevent non-specific binding, the slices were incubated with a normal goat serum sealer at room temperature for 20 min, and any excess liquid was discarded. Next, 50 μL of the primary antibody was added, and the slices were incubated overnight at 4°C. After washing twice with PBS for 5 min each time, the slices were incubated with a horseradish peroxidase-labeled secondary antibody for 1 h at room temperature. Following another two washes with PBS for 5 min each time, the slices were incubated with Streptavidin-Peroxidase at room temperature for 1 h. The slices were washed twice with PBS for 5 min each time before developing the DAB color for 5–10 min. The staining intensity was assessed under a microscope, considering cells with brown cytoplasm as positive cells. To stop the reaction, the slices were rinsed with cold water for 15 min. For further staining, the slices were briefly re-dyed with hematoxylin for 2 min, differentiated using hydrochloric acid and alcohol, and rinsed with cold water for 15 min. The slices were then conventionally dehydrated, made transparent, and sealed with a neutral gum drop next to the tissue, followed by covering with a cover glass. Finally, the slices were observed and photographed under a microscope.

### 5.15 Statistical analyses

Statistical analyses were performed using R statistical language (version 4.1.1). The Wilcoxon test and Kruskal–Wallis test were utilized for comparison between two and more than two groups, respectively. To draw the prognostic survival curve, the Kaplan–Meier plotter was employed, and the statistical significance was evaluated through the log-rank test. Spearman’s test was utilized for correlation analysis and calculation of correlation coefficient. The statistical significance level was set at *p* < 0.05 for all analyses.

## Data Availability

The original contributions presented in the study are included in the article/Supplementary Material, further inquiries can be directed to the corresponding authors.

## Supplementary material

Figure S1 Consensus matrixes of all ESCC samples for each k (k = 2–9), displaying the clustering stability using 1000 iterations of hierarchical clustering.

Figure S2 RT-qPCR results for the expression of PKP1 in ESCC cell lines cultured under normal and hypoxic conditions. *P<0.05, ns P >0.05.

## Abbreviations

ESCC, esophageal squamous carcinoma; EC, esophageal cancer; HPRs, hypoxia phenotype-related genes; TCGA, The Cancer Genome Atlas; GEO, Gene Expression Omnibus; FDR, false discovery rate; MSigDB, the molecular signatures database; DEGs, differentially expressed genes; PCA, principal component analysis; ssGSEA, single-sample gene-set enrichment analysis; GSVA, gene set variation analysis; GO, Gene Ontology; KEGG, Kyoto Encyclopedia of Genes and Genomes; PheWAS, phenome-wide association study; TME, tumor immune microenvironment.
