# Supplementary figures and images for: Identification and validation of prognostic and immunotherapeutic responses in esophageal squamous carcinoma based on hypoxia phenotype-related genes
Source: Front Pharmacol. 2024 Mar 7;15:1344317. doi: 10.3389/fphar.2024.1344317 (PMC10955338; doi:10.3389/fphar.2024.1344317)

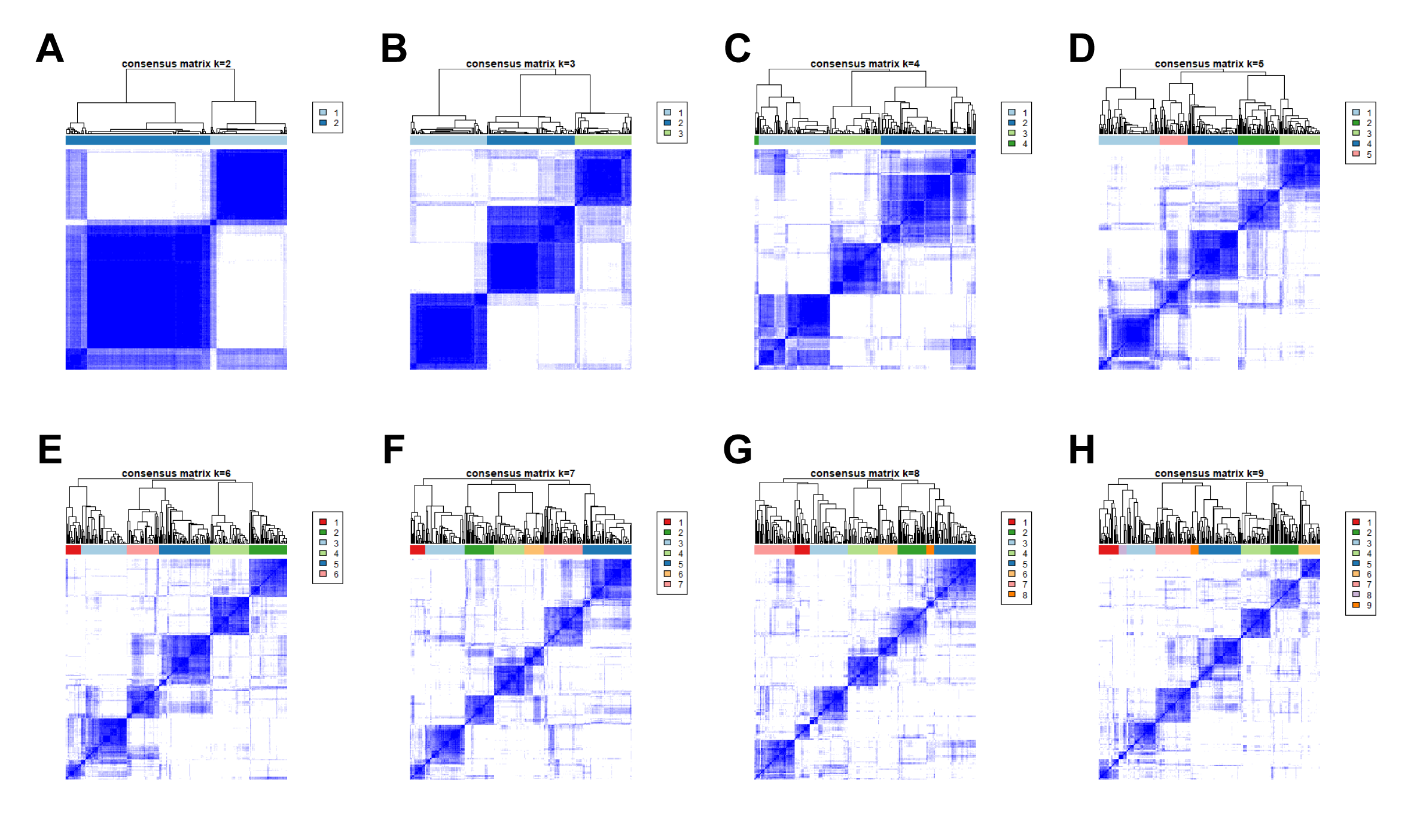

Supplement: Supplementary file 1 [file Image2.TIF]

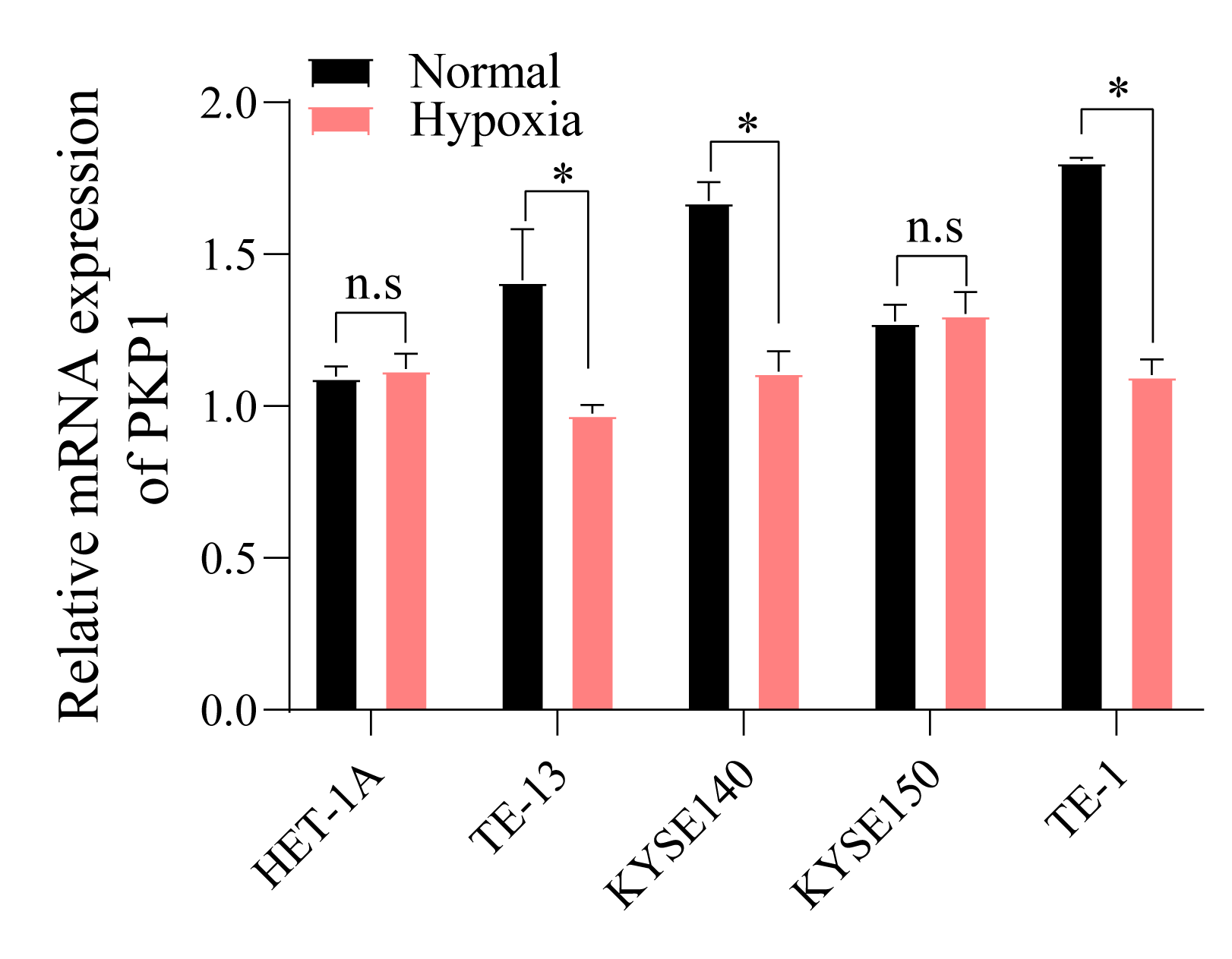

Supplement: Supplementary file 2 [file Image1.TIF]
